# Connectivity-based parcellation of the human frontal polar cortex

**DOI:** 10.1007/s00429-014-0809-6

**Published:** 2014-06-14

**Authors:** Massieh Moayedi, Tim V. Salomons, Katharine A. M. Dunlop, Jonathan Downar, Karen D. Davis

**Affiliations:** 1 Institute of Medical Science, University of Toronto, Toronto, M5S 1A8 Canada; 2 Department of Surgery, University of Toronto, Toronto, M5S 1A8 Canada; 3 Division of Brain, Imaging and Behaviour-Systems Neuroscience, Toronto Western Research Institute, Toronto Western Hospital, University Health Network, 399 Bathurst Street, Room MP14-306, Toronto, ON M5T 2S8 Canada; 4 Department of Psychiatry, University Health Network, Toronto, M5T 2S8 Canada; 5 Department of Neuroscience, Physiology and Pharmacology, University College London, London, WC1E 6BT UK; 6 School of Psychology and Clinical Language Science, University of Reading, Reading, RG6 6AL UK

**Keywords:** Diffusion MRI, BA10, White matter, Anatomy, Frontal lobe

## Abstract

The frontal pole corresponds to Brodmann area (BA) 10, the largest single architectonic area in the human frontal lobe. Generally, BA10 is thought to contain two or three subregions that subserve broad functions such as multitasking, social cognition, attention, and episodic memory. However, there is a substantial debate about the functional and structural heterogeneity of this large frontal region. Previous connectivity-based parcellation studies have identified two or three subregions in the human frontal pole. Here, we used diffusion tensor imaging to assess structural connectivity of BA10 in 35 healthy subjects and delineated subregions based on this connectivity. This allowed us to determine the correspondence of structurally based subregions with the scheme previously defined functionally. Three subregions could be defined in each subject. However, these three subregions were not spatially consistent between subjects. Therefore, we accepted a solution with two subregions that encompassed the lateral and medial frontal pole. We then examined resting-state functional connectivity of the two subregions and found significant differences between their connectivities. The medial cluster was connected to nodes of the default-mode network, which is implicated in internally focused, self-related thought, and social cognition. The lateral cluster was connected to nodes of the executive control network, associated with directed attention and working memory. These findings support the concept that there are two major anatomical subregions of the frontal pole related to differences in functional connectivity.

**Electronic supplementary material:**

The online version of this article (doi:10.1007/s00429-014-0809-6) contains supplementary material, which is available to authorized users.

## Introduction

**Fig. 1:**
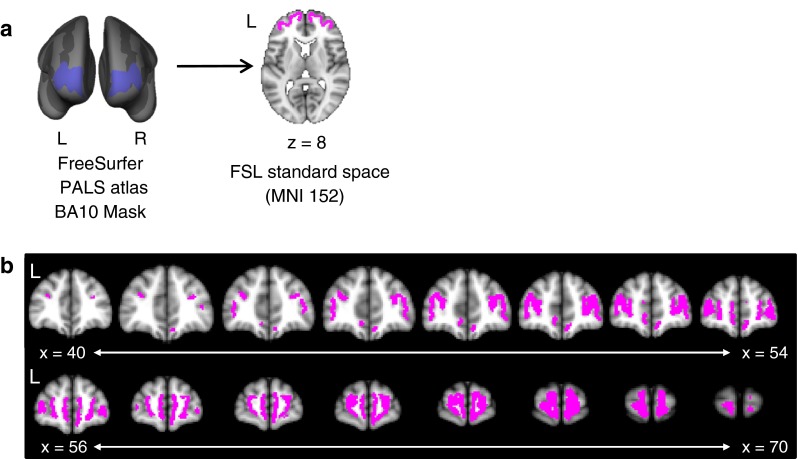
BA10 (frontal pole) mask from the PALS atlas (Van Essen 2005) from the FreeSurfer white matter surface. We converted this mask to the FSL standard space (MNI152) brain to perform the tractography-based parcellation analysis. The mask is displayed in **(a)** on the inflated brain surface in FreeSurfer (*left panel*) and the MNI152 brain in FSL (axial slice shown in the *right panel*). The span of the whole BA10 mask is shown in (**b**) across coronal slices

The most anterior portion of the primate brain is often designated as a single brain region of granular cortex, broadly defined as Brodmann area (BA) 10 in humans [and BA 12 in non-human primates, later reclassified as BA 10 by Walker (1940)], or the frontal polar cortex (FPC) (Fig. 1) (Barbas and Pandya 1989; Petrides et al. 2012). BA10 is a large region of the cortex and has mostly been associated with cognitive functions, such as multitasking, social cognition, attention, and episodic memory (Burgess et al. 2007; Gilbert et al. 2006, 2010b). These functions are supported by BA10 connectivity: in non-human primates, it is connected with prefrontal (lateral areas 8Ad, 8B, 46, 9/46, 45, and 47/12; medial areas 34, 32, and 9), orbitofrontal (areas: 11, 13, and 14), temporal (temporal pole, superior temporal gyrus and sulcus) and other brain regions (insula, posterior cingulate area 23, retrosplenial area 30, somatosensory-related parietal area 31) (Barbas et al. 1999; Barbas and Pandya 1989; Goulas et al. 2014; Petrides and Pandya 2011; Yeterian et al. 2012). These connections are primarily supported by the uncinate fasciculus and the extreme capsule fasciculus (Yeterian et al. 2012). The neuroanatomical features of BA10, such as cytoarchitecture, seem heterogeneous between human and non-human primates (Passingham and Wise 2012). Additionally, a distinguishing feature of the FPC is the high density of dendritic spines per cell, increased dentritic length, and a low density of cell bodies compared to comparable brain regions in humans (Jacobs et al. 1997, 2001). Together, these findings suggest that it is a highly integrative, supramodal brain region.

The human BA10 likely comprises structurally and functionally heterogeneous subregions based on cytoarchitectonic studies in humans. Connectivity-based parcellations seem to correspond to subregions identified cytoarchitectonically (Mars et al. 2011), and thus, may provide macrostructural evidence for subregions. The issue of BA10 parcellation has direct translational implications for the growing number of anatomically targeted neurological and psychiatric treatments: deep brain stimulation (DBS), epidural cortical stimulation (EpCS), repetitive transcranial magnetic stimulation (rTMS), and transcranial direct current stimulation (tDCS). Of particular importance for the treatment of major depression, several of these techniques target BA10 either directly or indirectly (Gustin et al. 2013; Iannetti et al. 2013), and therapeutic efficacy may hinge upon stimulation of an optimal subregion.

The macaque FPC consists of two cytoarchitectonically organized distinct regions: medial (10 m) and orbital (10o) (Carmichael and Price 1994). However, correspondence between FPC in non-human primates and the much larger BA10 in humans is uncertain (Semendeferi et al. 2001). A contentious issue is whether the human BA10 comprises two (FP1 and FP2) (Bludau et al. 2014) or three subregions [rostral (10r), medial (10 m), and polar (10p) (Ongur et al. 2003)].

Functional neuroimaging has identified subregions in BA10 that are consistent with cytoarchitectonically defined BA10 subdivisions (Gilbert et al. 2006, 2010b) and comprise either two or three functionally distinct subregions. A meta-analysis identified three BA10 subregions: the medial, rostral and lateral FPC (Gilbert et al. 2006), but other studies identified two subregions: the medial and the lateral FPC (Bludau et al. 2014; Charron and Koechlin 2010; Gilbert et al. 2010a; Koechlin 2011; Koechlin et al. 2000).

The medial FPC has been implicated in complex cognitive tasks, including social cognition (e.g., mentalizing) and relative reward monitoring (Boorman et al. 2009; Gilbert et al. 2006; Rushworth et al. 2011; Tsujimoto et al. 2010), whereas the rostral FPC has been associated with multitasking (Gilbert et al. 2006), and the lateral FPC with working and episodic memory, attention, cognitive branching, and task-switching (Boorman et al. 2009; Gilbert et al. 2006; Koechlin 2011; Rushworth et al. 2011). However, few studies have specifically investigated the differential roles of these putative functional subregions, including the contribution of adjacent anatomical regions (Mackey and Petrides 2010). Therefore, functional neuroimaging may not be the optimal approach to determine how many subregions exist in BA10.

The debate about whether there are two or three subregions in the FPC can be informed by examining the connectivity of this region (Beaulieu 2002, 2009). Diffusion-weighted imaging (DWI) can be used to delineate structural white matter connectivity in the brain and parcellate anatomical brain regions in a data-driven approach (Beckmann et al. 2009; Caspers et al. 2013; Johansen-Berg et al. 2004; Mars et al. 2011, 2012; Schubotz et al. 2010; Tomassini et al. 2007). A recent study used this method to determine the number of structural and functional subregions in the FPC (Liu et al. 2013) and identified three subregions. Another study, by Sallet and colleagues (2013), identified a single brain region, and a follow-up study by the same group (Neubert et al. 2014) identified two subregions using a more extensive FPC mask. Here, in parallel, we have assessed the correspondence between previously identified functional subregions and structural parcels at a population level in the FPC. Furthermore, we investigated whether the resting-state functional connectivity to the rest of the brain reflected the heterogeneity of the structural subregions we identified.

## Materials and methods

We investigated the structural connectivity of the FPC to identify subregions within this area with an approach that has been used to investigate the cingulate, parietal, and temporoparietal junction cortices (Beckmann et al. 2009; Johansen-Berg et al. 2004; Mars et al. 2011, 2012; Tomassini et al. 2007). To do so, our analysis involved three steps: (1) probabilistic tractography, seeded from the BA10 to the rest of the brain, (2) determination of the number of spatially consistent subregions using a *K*-means clustering algorithm of the tractographic data in each subject, and (3) characterization of the structural and functional connectivity of each of the subregions to the rest of the brain.

### Data acquisition

All subjects provided informed consent to procedures approved by the UHN Research Ethics Board. Diffusion-weighted images were acquired for 35 healthy subjects (17 women, 18 men; mean ± SD age 27.6 ± 6.41 years, range 18–39 years) on a 3T GE MRI (Signa HDx; maximum gradient strength = 40 mT/m, maximum slew rate = 150 T/m/s) system fitted with an eight-channel phased array head coil. Two sets of DWI data were acquired for each subject with 60 non-collinear, isotropic directions (repetition time = 17,000 ms, field of view: 23 × 23 cm, 96 × 96 matrix, 2.4 × 2.4 mm in-plane resolution, 2.4-mm-thick axial slices, with array spatial sensitivity encoding technique (ASSET) with a factor of 2; *b* = 1,000 s/mm^2^). Additionally, 10 non-diffusion-weighted scans (*b* = 0 s/mm^2^; b0) were acquired at the beginning of each run.

Also, a whole brain (180 sagittal slices, field of view: 25.6 × 25.6 cm^2^) high-resolution (256 × 256 matrix, 1 × 1 × 1 mm voxels) anatomical scan was also acquired for each subject using a 3D fast spoiled gradient-echo (FSPGR) sequence (flip angle 15˚, TE = min, TR = 7.8 ms).

T2*-weighted functional MRI scans with an echo-planar pulse imaging (EPI) sequence were also acquired for every subject (repetition time = 2,000 ms, echo time = 25 ms, axial slice thickness = 4 mm, field of view = 20 cm × 20 cm, 64 × 64 matrix, resulting in a voxel size of 3.125 × 3.125 × 4 mm^3^, 150 volumes). For the 5-min task-free scan, subjects were instructed to lie still, clear their thoughts and “not to think about anything in particular”, with their eyes closed.

### Anatomical parcellation using DWI

#### Seed region definition

The selection of the mask to define the frontal pole is crucial to the outcome of the parcellation method. In the current study, we used the Brodmann parcellation scheme, specifically region BA10, which corresponds to the frontal polar cortex. This decision was based on the common use of the term BA10 to describe frontal polar findings.

The bilateral BA10 were determined using a cortical surface parcellation atlas included in the FreeSurfer software package version 5.0.0 (http://surfer.nmr.mgh.harvard.edu/). We used the PALS (http://brainvis.wustl.edu/wiki/index.php/Caret:Atlases; Van Essen 2005) Brodmann area parcellation atlas to define a mask for BA10 for each hemisphere in FreeSurfer standard space (fsaverage). Importantly, the masks were defined on the surface of white matter, i.e. on the border of white and grey matter in the brain (see Fig. 1). These masks (one for each hemisphere) were then transformed to FMRIB Software Library (FSL version 4.1.2; http://www.fmrib.ox.ac.uk/fsl/; Smith et al. 2004) standard space (MNI152) for analysis (see Fig. 1). The BA10 mask was transformed to individual space using the linear registration tool (FLIRT) implemented in FSL, using 12 degrees of freedom (Jenkinson et al. 2002), and visually checked for aberrant registration. All registrations were satisfactory upon visual inspection.

#### Anatomical parcellation using probabilistic tractography

**Fig. 2:**
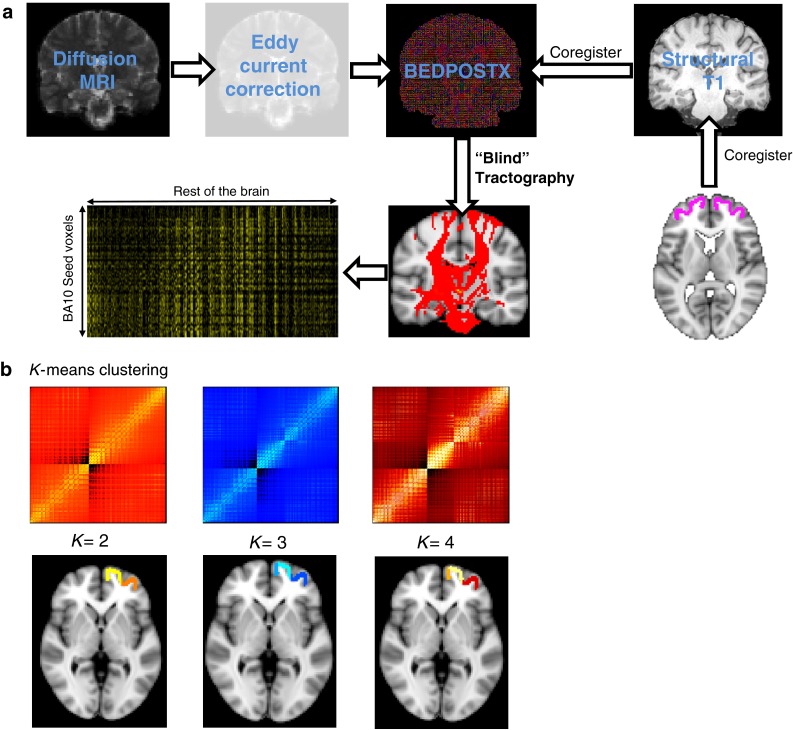
Preprocessing and analysis pipeline for diffusion-weighted parcellation of BA10. **a** Diffusion data are eddy-current corrected and registered to the B0 image. Next probability density functions on up to two principal fibre directions were estimated at each voxel in the brain using the Bayesian estimation of diffusion parameters obtained using sampling techniques toolbox (BEDPOSTX) implemented in FSL. Diffusion data were also co-registered to a T1-weighted anatomical scan. Next, probabilistic tractography was run from every voxel in the BA10 seed (registered to each subject’s diffusion space) to the rest of the brain, in a lower resolution brain (voxel size 5 × 5 × 5 mm). This resulted in a matrix of the probability of connection of every voxel in the seed to every other voxel in the brain. **b** These matrices have been cross-correlated and clustered according to a *K*-factor, which represents the number of clusters output by the algorithm. The parcellations are shown on the T1-weighted MNI standard brain in FSL

A summary of these methods can be found in Fig. 2. DWI data were preprocessed using tools from FDT, part of FSL. Motion and eddy-current correction were performed using affine registration of all volumes to a target b0 volume—the second acquired volume. Probability density functions on up to two principal fibre directions were estimated at each voxel in the brain using the Bayesian estimation of diffusion parameters obtained using sampling techniques toolbox (BEDPOSTX; Behrens et al. 2007) implemented in FSL. We then used multi-fibre tractography (maximum number of steps = 2,000, curvature threshold = 0.2) and drew 5,000 samples from each voxel in the BA10 mask to every brain voxel (downsampled to 5-mm isotropic voxels). The number of samples that reach a voxel in the brain is considered the connectivity of the seed to that voxel.

We parcellated BA10 using previously described methods (Beckmann et al. 2009; Johansen-Berg et al. 2004; Mars et al. 2011, 2012) in the “ccops” toolbox implemented in FSL. In brief: for each subject, a connectivity matrix between BA10 voxels and each voxel of the downsampled (5 mm isotropic) brain voxel was derived (Johansen-Berg et al. 2004). The matrices consist of rows indicating each BA10 voxel, and columns representing each voxel in the rest of the brain. The values in each element of the matrix represent a proxy measure of the connectivity value of the BA10 voxel and the brain voxel (i.e., the probability of connection of the two voxels). We then generated a symmetric cross-correlation matrix of dimensions equal to the number of seed voxels by the number of seed mask voxels. The (*i*, *j*)th element within the matrix represents the correlation between the connectivity profile of voxel *i* and the connectivity profile voxel *j*. We then permuted the rows of the cross-correlation matrix using a *K*-means clustering segmentation algorithm, implemented in the ccops toolbox in FSL, for automated clustering to define *K* different clusters. This algorithm randomly selects a starting point in the matrix to cluster the voxels in the seed (i.e., the voxels within the BA10 mask) that have similar connectivity profiles (i.e., connectivity values to the rest of the voxels in the brain). It is possible that two separate regions cluster as a single cluster because of their interconnectivity (c.f. the similarity of their connectivity to the rest of the brain). Therefore, we included a scaled Euclidian distance matrix to the cross-correlation matrix as implemented in FSL (Tomassini et al. 2007). We used a weak distance constraint of 0.2, as has been previously used (Beckmann et al. 2009; Mars et al. 2011, 2012; Tomassini et al. 2007). This results in clusters of spatially contiguous voxels, although the border between clusters is guided by connectivity to voxels in the rest of the brain.

The *K*-means clustering algorithm requires us to set the number of clusters (*K*) that are formed. Previous work has suggested that there are either two or three subregions in BA10 (Charron and Koechlin 2010; Gilbert et al. 2006, 2010a; Koechlin 2011; Koechlin et al. 2000; Ongur et al. 2003; Tsujimoto et al. 2010). Therefore, we used an iterative method to determine the number of stable clusters (i.e., spatially consistent) that can be formed across the study population (Beckmann et al. 2009; Mars et al. 2011, 2012). Specifically, we tested for *K* values of 2, 3 and 4, and determined the highest *K* value that was able to obtain spatially consistent clusters across all subjects, and the subregions created a continuous area of cortex.

#### Probabilistic tractography from resultant BA10 subregions

To qualitatively demonstrate the differential structural connectivity of the subregions, we performed probabilistic tractography with the same parameters as above from each cluster for each subject. The tractography was unrestrained by any masks and was run along the main and secondary fibre directions, as determined by the BEDPOSTX algorithm, to the rest of the brain. The target mask included the whole brain (limited by the grey matter–pial layer boundary). The resulting tractograms were thresholded at 5,000 samples to demonstrate regions of differential connectivity. The tractograms were then binarised and summed to make a group probabilistic tractogram for each cluster (Fig. 5).

### Resting-state functional connectivity

#### Data analysis

Prior to analysis, the first four volumes of the resting-state fMRI data were deleted to allow signal equilibration. The data were subsequently preprocessed and analyzed in the Conn toolbox v.13 (http://www.nitrc.org/projects/conn; Whitfield-Gabrieli and Nieto-Castanon 2012), implemented in Matlab v.7.14.0 (Mathworks, Natick, MA, USA). First, the toolbox uses tools in SPM8 (Wellcome Department of Imaging Neuroscience, London, UK; http://www.fil.ion.ucl.ac.uk/spm) to spatially preprocess each subject’s functional data. These steps include realignment (motion correction), coregistration to a structural T1 image, normalization to the MNI standard brain, and spatial smoothing (6-mm FWHM Gaussian filter). Additionally, in the Conn toolbox, subjects’ anatomical T1-weighted images are segmented for grey matter, white matter and cerebrospinal fluid (CSF), and eroded (one-voxel erosion; 2 mm isotropic voxel size) to later remove temporal confounds related to these tissue types (see below). The data were also temporally preprocessed to control for other potential confounds and to restrict the analysis to frequencies of interest (<0.1 Hz). These steps include using linear regression to remove potential sources of noise, including estimated subject motion parameters (3 translation components and 3 rotation components), BOLD signals in white matter and CSF areas. The additional white matter and CSF covariates are included using the anatomical component-based noise correction method (aCompCor; Behzadi et al. 2007). We set the algorithm to compute five orthogonal timeseries (components) for white matter and five orthogonal timeseries (components) for CSF in each subject. The residual BOLD image is band-pass filtered between 0.01 and 0.1 Hz.

We performed seed-based resting-state functional connectivity (Biswal et al. 1995; Fox et al. 2005; Greicius et al. 2003; Taylor et al. 2009) between the BA10 subregions identified with probabilistic tractography (see above and Fig. 4a) for each hemisphere and the rest of the brain. The first-level bivariate correlation maps were calculated between the seeds and the rest of the brain. These correlation values are then Fisher transformed to normalized *Z*-statistics for second-level comparisons. We compared the functional connectivity of the two (lateral and medial) seeds within each hemisphere. Second-level group-level random-effects analysis was thresholded at *p* < 0.05 corrected for multiple comparisons with family-wise error correction with an extent threshold of 8 voxels. The final results were displayed on the FSL standard brain (MNI152_T1_2mm_brain.nii.gz).

## Results

### Anatomical parcellation using probabilistic tractography

**Fig. 3:**
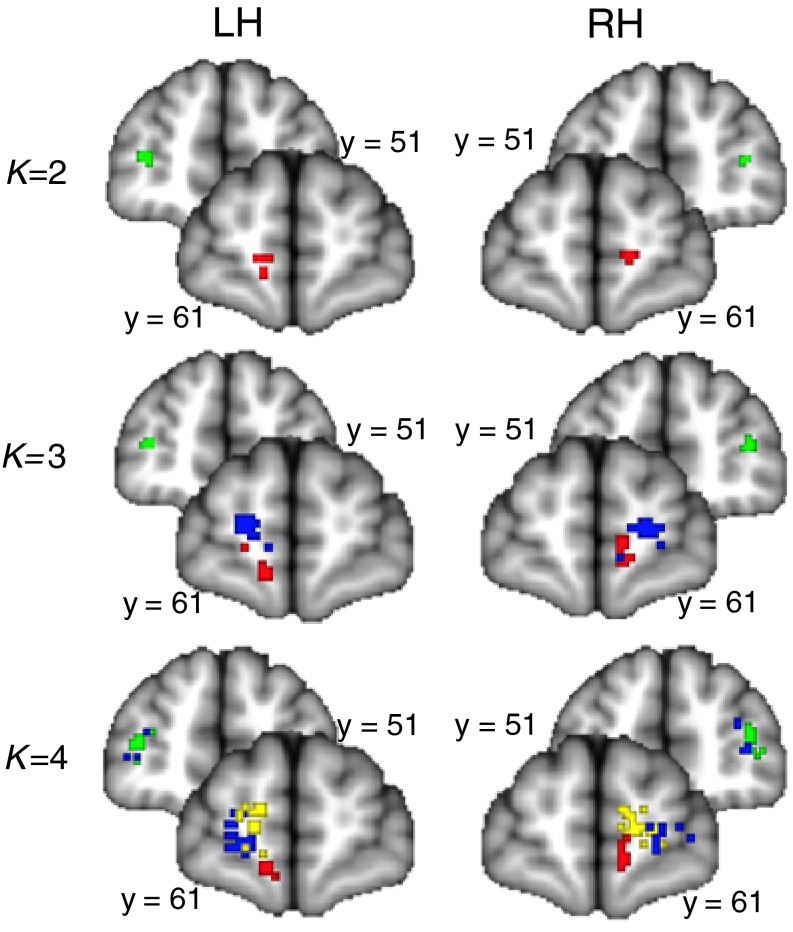
Individual-level results of parcellation solutions of the bilateral BA10 for each of the 35 subjects plotted on the MNI152 standard brain. The analysis was run separately for each hemisphere. Each point represents the centre-of-gravity of each cluster in each subject—there are 35 points in each image, although this may not be clear due to overlap. The two-cluster solution (*K* = 2) showed a lateral (green) and medial (*red*) clusters. The three-cluster solution (*K* = 3) showed the lateral (*green*), medial (*red*) and rostral (*blue*) clusters. The four-cluster solution (*K* = 4) showed a lateral (*green*), medial (*red*), rostral (*blue*) and dorso-medial rostral (*yellow*) cluster. Note the consistency in the two-cluster solution

**Fig. 4:**
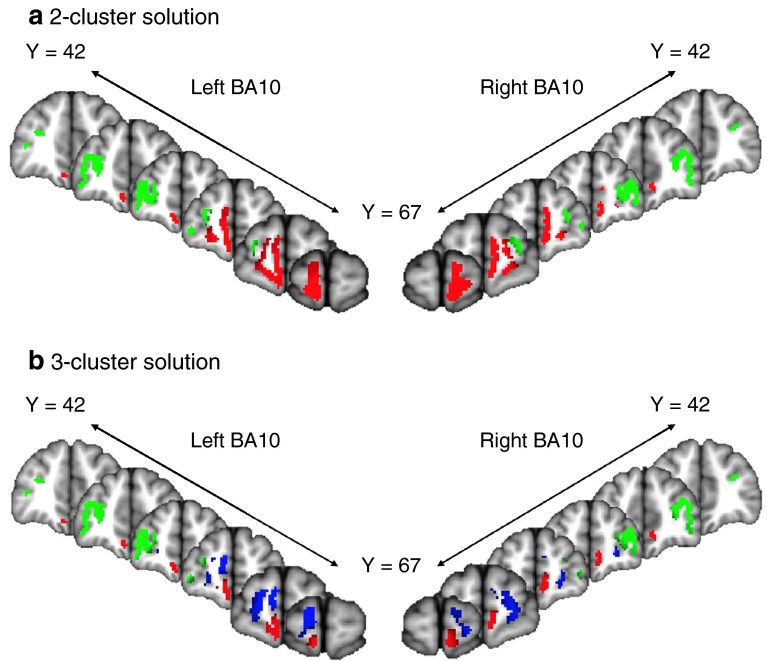
Group-level results of parcellation solutions for the bilateral BA10 shown on the MNI152 brain. The group parcellations images were created based on areas of the frontal pole that showed overlap across subjects. **a** In the two-cluster solution, the medial cluster is shown in *red* and the lateral cluster is shown in *green*. The images are thresholded at 75 % (26/35) of all subjects. The left medial had a centre of gravity (COG all coordinates are in MNI space) at (−6, 58, −12), and the right medial cluster had a COG at (8, 58, −6). The left lateral cluster had a COG at (−34, 50, 10), and the right lateral had a COG at (36, 50, 14). **b** In the three-cluster solution, the medial cluster is shown in *red*, the rostral cluster is shown in blue, and the lateral cluster is shown in *green*. The images are thresholded at 75 % (26/35) of all subjects. The medial cluster had a COG at (−12, 64, 12) on the left, and at (20, 62, 4) on the right. The rostral cluster had a COG at (−6, 58, −12) on the left and the COG at (8, 58, −6) on the right. The lateral cluster had a COG at (−34, 50, 10) on the left, and the COG at (36, 50, 14) on the right

The first aim of this study was to parcellate the human frontal polar cortex into distinct subregions based on their structural white matter connectivity, using probabilistic tractography. The clusters were formed based on a *K*-means clustering algorithm. We used an iterative process, guided by previous studies that suggest that there are either two or three anatomically distinct subregions in the FPC. We performed the clustering algorithm on the FPC with an increasing number of clusters (2, 3, and 4) and determined the largest value for *K* where the clusters remained consistent amongst all 35 subjects (Beckmann et al. 2009; Mars et al. 2011, 2012). Each individual’s parcellation results are shown in Supplemental Fig. 1, and the centre-of-gravity of each individual parcellation results is shown in Fig. 3. The two-cluster solution resulted in a medial and a lateral cluster (Figs. 3, 4a). Bilaterally, the medial clusters spanned the ventral portion of the medial frontal gyrus, superior to the straight gyrus (*gyrus rectus*), and anterior to the cingulate sulcus. Bilaterally, the lateral clusters spanned the middle frontal gyrus, between the superior frontal sulcus and the inferior frontal sulcus. All four of these clusters were consistent amongst all subjects. The three-cluster solution resulted in a medial, a rostral and a lateral cluster (see Figs. 3, 4b). In this solution, the medial cluster spanned medial frontal gyrus superior to the straight gyrus, bilaterally. The lateral cluster spanned the anterior portions of the inferior frontal and middle frontal gyri, bilaterally. The rostral clusters spanned the dorsal portion of medial frontal gyrus and the rostral superior frontal gyrus, medial to the superior frontal sulcus, bilaterally. The four-cluster solution did not produce a consistent map, which is demonstrated by overlap between the centre-of-gravity of separate clusters between subjects (Fig. 3). This demonstrates that the clusters are not spatially consistent across subjects. For example, the right lateral cluster does not parcellate into two subregions in 6/35 subjects, and there is substantial overlap between group maps of the clusters (Fig. 3). Based on these parcellations, we determined that the two-cluster and three-cluster solutions both produced consistent clusters amongst all subjects, and could provide plausible and robust solutions for subregions within the FPC. The four-cluster solution was not pursued further.

### BA10 subregion white matter connectivity

**Fig. 5:**
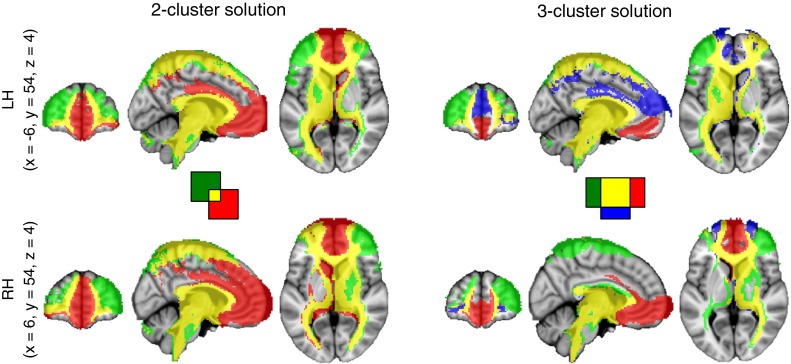
Group-level tractograms for each of the BA10 clusters to the rest of the brain. The *red* tracts are seeded from the medial cluster, the *green* tracts are seeded from the lateral cluster, and the *blue* tracts (in the three-cluster solution) are seeded from the rostral cluster. The *yellow* tracts represent the overlap of the tracts. The individual tractograms were thresholded at 5,000 samples. Each subject’s tractogram was then overlaid onto the MNI152 brain to make a group tractogram. The image displayed consists of a group map of tracts that overlap in at least 50 % (17/35) subjects

We next created a group map for each cluster based on the probabilistic tractography parcellation results described above (Fig. 5). Differences in BA10 connectivity were assessed based on regions where the tractograms of the clusters showed no overlap at a specific threshold.

For the two-cluster solution, the lateral cluster, but not the medial cluster, was connected to lateral prefrontal cortex (PFC BA 46, 9, 6 and 8), the ipsilateral pallidum and putamen, and the pons. The medial cluster, but not the lateral cluster, was connected to the medial PFC, the cingulate cortex, the orbitofrontal cortex and the contralateral caudate nucleus.

For the three-cluster solution, all three clusters had widespread connections. The parcels did have differential connectivity, albeit with some overlap. For example, tracts from the medial cluster uniquely projected to medial frontal brain regions, including the medial PFC (BA10), orbitofrontal (BA11) and anterior cingulate (BA 24/32) and subgenual cingulate (BA 25) cortices. The left rostral cluster uniquely projected to the anterior, mid and posterior regions of the cingulate cortex (BA 24/32, 23 and 31) the dorsomedial PFC (BA 8, 9 and 6). In the right hemisphere, the rostral cluster did not uniquely project to any brain regions. The lateral cluster projected to lateral frontal (BA 8, 9 and 6) and parietal cortical regions, as well as subcortical regions.

In the three-cluster solution, the medial and rostral clusters are subdivisions of the medial cluster of the two-cluster solution. Given that the rostral cluster of the three-cluster solution did not have a unique pattern of structural connectivity distinct from the medial cluster, the three-cluster solution was not pursued further. Instead, the two-cluster solution was used for the purposes of resting-state functional connectivity analysis.

### BA10 subregion resting-state functional connectivity

**Fig. 6:**
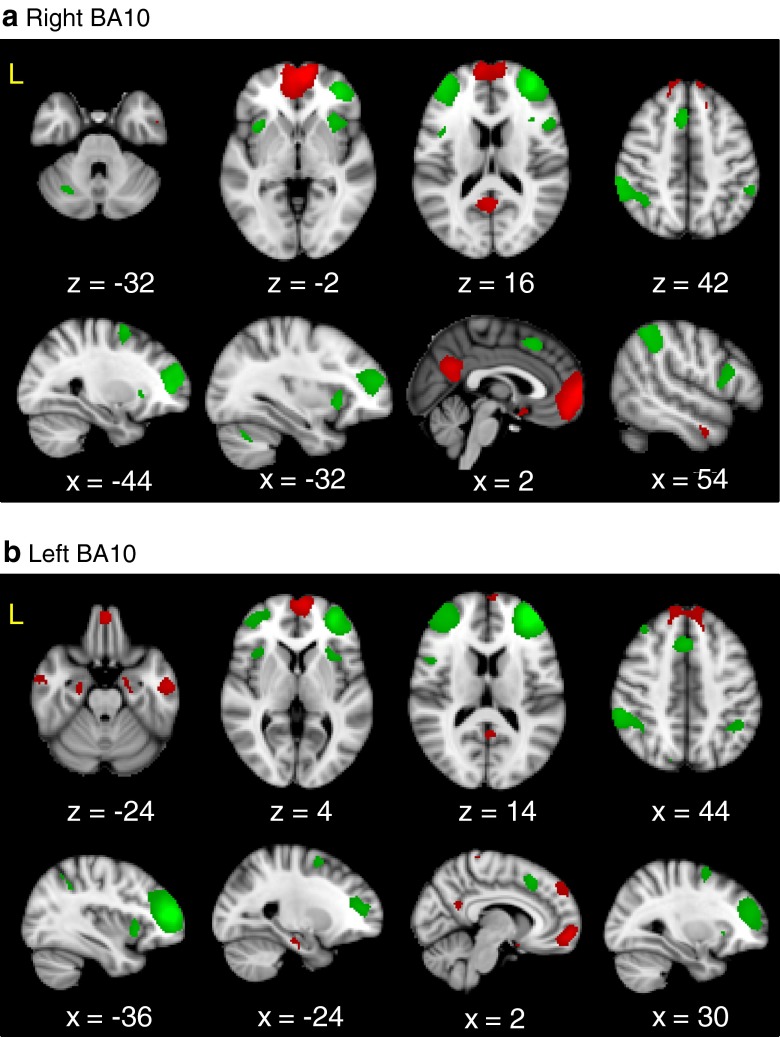
Difference in resting-state connectivity of the BA10 clusters from the two-cluster solution in (**a**) the right hemisphere BA10 clusters and (**b**) the left hemisphere BA10 clusters. Regions significantly more connected to the medial cluster are shown in *red* and regions significantly more connected to the lateral cluster are shown in *green*

It is critical to note that structural connectivity may or may not be reflected in functional connectivity. The individual nodes within networks of brain regions may lack direct structural connectivity identified with diffusion-weighted tractography (i.e. there may be indirect, polysynaptic connections between regions, or this lack of connectivity may be due to the limitations of tractography in tracing long distance connections), and yet the BOLD activity within these nodes may still be correlated, with the nodes co-activating in a reliable fashion to subserve a common function. Thus, the specific set of other brain regions with activity correlating with each of the FPC subregions could conceivably provide additional useful information regarding their respective functions. Therefore, we used resting-state fMRI to investigate the functional connectivity of the medial and lateral subregions identified in the two-cluster solution (Fig. 6).

We found that the seeds in the two-cluster solution activated two distinct networks of regions in resting-state fMRI. Bilaterally, the medial clusters were significantly more functionally connected to nodes of the default-mode network (see Fig. 6): namely, the bilateral medial PFC, the bilateral precuneus/posterior cingulate cortex, the ipsilateral lateral occipital cortex, the bilateral parahippocampal gyri, the bilateral subgenual cingulate cortex, the bilateral middle temporal gyrus. Conversely, the bilateral lateral clusters were functionally connected to nodes of the executive control network, including the bilateral supplementary motor area, the ventrolateral premotor cortex, the lateral parietal area, the dorsolateral PFC (dlPFC) and the bilateral anterior insula. There were no sex differences in the connectivity of the medial or lateral clusters.

## Discussion

The aim of the current study was to investigate whether there are discernible structural subregions in the FPC based on white matter connectivity profiles. We used a data-driven approach based on probabilistic tractography to determine the connectivity of every voxel within BA10, and a clustering algorithm to parcellate the BA10 into subregions. Based on structural connectivity, we found two solutions that were reliably reproducible across 35 subjects: a two-cluster and a three-cluster solution. The two-cluster solution comprised a medial and a lateral cluster, whereas the three-cluster solution further divided the medial cluster into a more ventral and a more dorsal cluster (which we termed the rostral FPC). Structural connectivity of these clusters revealed that at the population level the two-cluster solution was more consistent than the three-cluster solution. Specifically, there were no unique tracts in half of the subjects for the rostral cluster in the right hemisphere in the three-cluster solution, whereas the two-cluster solution showed clear differences.

The structural and functional heterogeneity of the FPC has been previously explored using a variety of methodologies (Bludau et al. 2014; Gilbert et al. 2006, 2007, 2010b; Koechlin et al. 2000; Liu et al. 2013; Neubert et al. 2014; Ongur et al. 2003; Ongur and Price 2000; Sallet et al. 2013; Semendeferi et al. 2011). However, to date, there is no consensus on the number of subregions in FPC. For instance, histological studies in non-human primates have revealed two distinct regions in the FPC, based on cytoarchitecture (Carmichael and Price 1994). The extent to which this cytoarchitectonic parcellation is applicable to humans, however, is subject to further investigation (Passingham 2009). Comparative anatomical studies have demonstrated that the human BA10 is proportionately much larger than the analogous structure in other primates (Semendeferi et al. 2001). Also, the spatial organization of cellular columns in human BA10 differs from BA10 in great apes, including cortical column organization that allows for more columnar interconnectivity (Semendeferi et al. 2011). Nonetheless, the study by Bludau et al. (2014) revealed two BA10 subregions in humans, similar to the non-human primate analogue of BA10. However, these subregions show different anatomic features and functions—especially the lateral FPC, which seems to be unique to humans (Neubert et al. 2014).

Diffusion-weighted tractography represents a reliable and valid methodology for investigating the neuroanatomical structure of the brain (Anwander et al. 2007; Beckmann et al. 2009; Eickhoff et al. 2010; Johansen-Berg et al. 2005; Klein et al. 2007, 2009; Mars et al. 2011, 2012; Schubotz et al. 2010; Tomassini et al. 2007). The extrinsic connections of a brain region constrain its function, and so the patterns of connectivity within a region can be used to discern functionally distinct areas (Averbeck et al. 2009; Mars et al. 2011; Passingham et al. 2002). Furthermore, tractographic findings in humans have been validated by comparing and correlating to tract-tracing and tractographic studies in non-human primates (Croxson et al. 2005; Dauguet et al. 2007; Dyrby et al. 2007; Mars et al. 2011). Three studies exploit this method to study the number of structural and functional subregions in the human FPC (Liu et al. 2013; Neubert et al. 2014; Sallet et al. 2013). The study by Sallet et al. (2013) identified a single BA10 region, whereas the study Neubert et al. (2014) identified two subregions, and the study by Liu and colleagues (2013) identified three distinct subregions in the FPC. It is noteworthy, however, that the differences between the findings in the Sallet et al. (2013) and Neubert et al. (2014) studies can be attributed to differences in the region-of-interest they investigated—the former study investigates a more dorsal FPC, whereas the latter study investigated a region of the FPC similar to the one in the current study.

Using similar methods in a larger sample than all three studies, our data confirm that there are both structurally and functionally discernible subregions in the FPC in humans. Our three-cluster solution closely resembles the findings of Liu and colleagues (2013) in that we also identified a rostral, medial and lateral cluster that spanned the same anatomical regions. However, we found that, in line with the findings in the Bludau et al. (2014) and Neubert et al. (2014) studies, the most reliable pattern of differential structural connectivity emerged from a two-cluster solution dividing the FPC into a lateral and a medial subregion, and that the rostral cluster in the three-cluster solution did not have unique population-level (in 50 % of subjects) structural connectivity in the right hemisphere. In the two-cluster solution, the lateral cluster was structurally connected to lateral PFC areas and associated striatal structures and the medial cluster was connected to medial and ventral PFC areas.

Our aim was to establish a population-based mask of the FPC based on connectivity-based parcellation. As noted by Caspers et al. (2013), this method can provide a framework to study individual differences. In the current study, we accepted the two-cluster solution as the most consistent solution for a population-based map of the FPC. However, our three-cluster and four-cluster solutions highlight individual differences in brain anatomy as evidenced by the variability in the spatial distribution of the resulting clusters. It is also possible that the mask, in some subjects, included medial BA 11, an adjacent brain region ventral to the medial FPC (Mackey and Petrides 2010). These individual differences may underlie differential behavioural strategies and functional heterogeneity, and may therefore be of additional interest in studies of between-subject heterogeneity rather than average behaviour and function (Mueller et al. 2013).

A recent study by Catani and colleagues (2012) used high-resolution diffusion imaging to perform tractographic-based dissections of several white matter tracts. This study demonstrated that two subregions of the FPC, corresponding closely to the subregions identified in the present study, have differential anatomical white matter connectivity. Specifically, the medial subregion of Catani et al. (2012) was largely connected via the frontal superior longitudinal tracts, while the lateral subregion was mostly connected via the frontal inferior longitudinal tracts. The connections of these tracts are consistent with our finding that the lateral cluster is structurally and functionally connected to lateral brain regions, and that the medial cluster is structurally and functionally connected to subcortical and medial brain regions. Furthermore, they demonstrated that a prominent *U*-shaped tract, the fronto-marginal tract, connects the lateral and medial subregions. These data, assessed with a qualitative high-resolution diffusion tractography, reflect our probabilistic tractography findings by establishing that the two subregions of the FPC have differential white matter connectivity, and provide detailed evidence that both local, short fibre connections and long association tracts contribute to the structural heterogeneity of the FPC.

In addition to their convergence with findings from in vivo tract tracing studies, the results of the present study are consistent with previous functional neuroimaging studies that demonstrate different subregions in the FPC that participate in different types of cognition and coactivate with different functional cortical networks. Gilbert and colleagues (2006, 2010b) demonstrated that a region of rostral (anterior polar) FPC, closely corresponding to our medial FPC region, was related to multitasking. A more lateral subregion, corresponding to our lateral FPC, was related to episodic memory retrieval, while a more medial subregion was related to social cognition. A study of brain areas that co-activated with the medial and lateral subregions across various tasks (Gilbert et al. 2010a) demonstrated that the medial subregion was co-activated with nodes of the default-mode network, including the PCC and the hippocampus, whereas the lateral subregion was connected to the midcingulate cortex/supplementary motor area (MCC/SMA), insula and the lateral parietal cortices. Another co-activation meta-analysis study by Bludau and colleagues (2014) corroborates the findings from the Gilbert (2010a) study. Interestingly, it has been proposed that anterior ventral medial PFC (corresponding to the medial FPC cluster of the present study) computes the value of choices (Smith et al. 2010). Furthermore, De Martino and colleagues (2012) interpreted the activity in the medial FPC and the PCC/PCu as representing the difference in value of two options, with the lateral subregion encoding the confidence of that choice and the functional connectivity of these regions modulating the confidence of that choice. The FPC is also implicated in the tracking of long-term goals. For example, the medial FPC is associated with tracking internally specified goals, whereas the lateral FPC is associated with tracking externally specified goals (Koechlin et al. 2000). These concepts suggest that anatomically distinct regions, as identified by white matter parcellation, have distinct and complementary roles in metacognition. Specifically, the medial FPC would thus have a role in tracking and evaluating competing stimuli by comparing stimulus information to previously stored information, by retrieving related memories (Euston et al. 2012), and the lateral FP would function to select and initiate the appropriate behaviour based on feedback from the medial FP.

Previous parcellations of the human BA10 have been based primarily on functional data. However, we wished to establish the correspondence between functional and structural parcellation and to determine whether these regions have distinct functions. Therefore, we used resting-state fMRI to test whether the anatomically derived subregions differ in functional connectivity. Similarly to the Neubert et al. (2014) study, in the two-cluster solution, we found that the medial cluster was more functionally connected to the medial PFC, the PCC and the temporal lobe. These are in line with tracing studies that have identified dense connections with the medial premotor regions (e.g., cingulate motor areas) and temporal regions, including the temporal pole, superior temporal and parahippocampal gyri (Barbas and Pandya 1989; Passingham and Wise 2012; Petrides and Pandya 2007). The lateral cluster is more functionally connected to nodes of the executive control network, including the dlPFC and the SMA (Seeley et al. 2007; Weissman-Fogel et al. 2010)—which is more active during externally rather than internally focused cognition. Crucially, these functional connectivites and the required underlying anatomical connections are absent in non-human primates (Neubert et al. 2014; Saleem et al. 2013), which suggest that the human BA10 has a unique structure and function. Additionally, our results are in line with previous research suggesting distinct recruitment of medial and lateral FPC for internally versus externally specified goals (Koechlin et al. 2000). Thus, functional connectivity is consistent with structural connectivity in demonstrating two distinct regions of FPC, differentially linked to cortical networks for internally versus externally focused cognitive processes.

A reliable functional parcellation of BA10 may also have important clinical implications for neurostimulation therapies of psychiatric illnesses, such as major depressive disorder (MDD). The oldest such treatment, electroconvulsive therapy (ECT), conventionally places electrodes over the lateral frontotemporal or parietal cortex. Although the effects of ECT on brain activity are widespread, the effectiveness of the treatment correlates best to the degree of reduction in frontopolar metabolism (Henderson et al. 2013; Jensen et al. 1994), suggesting that the FPC could be a more effective stimulation target. Notably, a recently developed variant of electroconvulsive therapy, known as focal electrically administered seizure therapy (FEAST), targets the frontal pole (Iannetti et al. 2013). The connectivity of the medial BA10 subregion suggests that this area could represent an optimal target to modulate pathological forms of rumination, self-reflection, and default-mode activity seen in MDD (Davis and Moayedi 2012; Mur et al. 2009). A milder, nonconvulsive form of electrical stimulation, tDCS, has also shown promising but inconsistent efficacy for MDD using a target in the dlPFC (Brunoni et al. 2013; Liang et al. 2013). The medial BA10 subregion could potentially serve as a more effective stimulation target for future tDCS studies. Likewise, noninvasive rTMS for MDD conventionally targets the dlPFC, although other targets have been proposed, including the FPC (Downar and Daskalakis 2012). As rTMS offers more precise focal stimulation than external electrodes, our results (namely, the identification of distinct medial and lateral BA10 parcels) may be particularly helpful in informing the optimal placement of the stimulation coil in future studies of FPC-rTMS in MDD. Finally, deep brain stimulation DBS and EpCS have been used to treat MDD (Kennedy et al. 2012; Nahas et al. 2010; Treede et al. 1999). Our results here could help to inform the optimal placement of DBS electrodes within the white matter tracts of the subcallosal cingulate gyrus (Geisler et al. 1958) or the medial forebrain bundle (Gustin et al. 2013). They could also help to inform the choice of new stimulation targets for EpCS, which until now has only been applied to prefrontal regions posterior to BA10 (Youssef et al. 2014). Specifically, the medial BA10 subregion and its associated white matter tracts may represent promising targets for EpCS and DBS, respectively.

In summary, the present study found that human FPC is structurally and functionally heterogeneous, with a reliable two-cluster separation between a medial cluster coactive with internally directed or default-mode networks and a lateral cluster coactive with externally directed or central executive networks in the resting brain. A more subtle separation of the medial cluster into a medial and a rostral subcluster, which has previously been reported (Liu et al. 2013), was less consistent across hemispheres and subjects in our study sample, with less distinct patterns of projection between subclusters in at least 50 % of subjects. In the future, more detailed investigations of the FPC using high-field MRI and histological methods in a larger sample will help to clarify the typical and the variant features of FPC anatomy across human individuals.

## Electronic supplementary material

Below is the link to the electronic supplementary material.

Supplementary material 1 (PDF 395 kb)
