# Supplementary material for: Connectivity-based parcellation of the human frontal polar cortex
Source: Brain Struct Funct. 2014 Jun 14;220(5):2603–16. doi: 10.1007/s00429-014-0809-6 (PMC4549383; doi:10.1007/s00429-014-0809-6)
Supplement: Supplementary file 1 — Supplementary material 1 (PDF 395 kb) [file 429_2014_809_MOESM1_ESM.pdf]

Supplementary Materials for:

Connectivity-based Parcellation of the Human Frontal Polar Cortex

Massieh Moayedi[1,3], Tim V. Salomons[3,4], Katharine A. M. Dunlop[4], Jonathan Downar[4], and Karen D. Davis[1,2,3]

[1]Institute of Medical Science, [2]Department of Surgery, University of Toronto, Toronto, Canada, M5S 1A8; [3]Division of Brain, Imaging and Behaviour - Systems Neuroscience, Toronto Western Research Institute, and [4]Department of Psychiatry, University Health Network, Toronto, Canada, M5T 2S8

Correspondence to:

Karen D. Davis, Ph.D.
Division of Brain, Imaging and Behaviour – Systems Neuroscience
Toronto Western Research Institute
Toronto Western Hospital,
University Health Network
399 Bathurst Street, Room MP14-306
Toronto, Ontario, Canada M5T 2S8
(416) 603-5662 ph; (416) 603-5745 fax
Email: kdavis@uhnres.utoronto.ca

CONTENTS:

Supplementary Figure 1: Individual parcellation results

Supplementary Table 1: Functional connectivity of 2-cluster solution

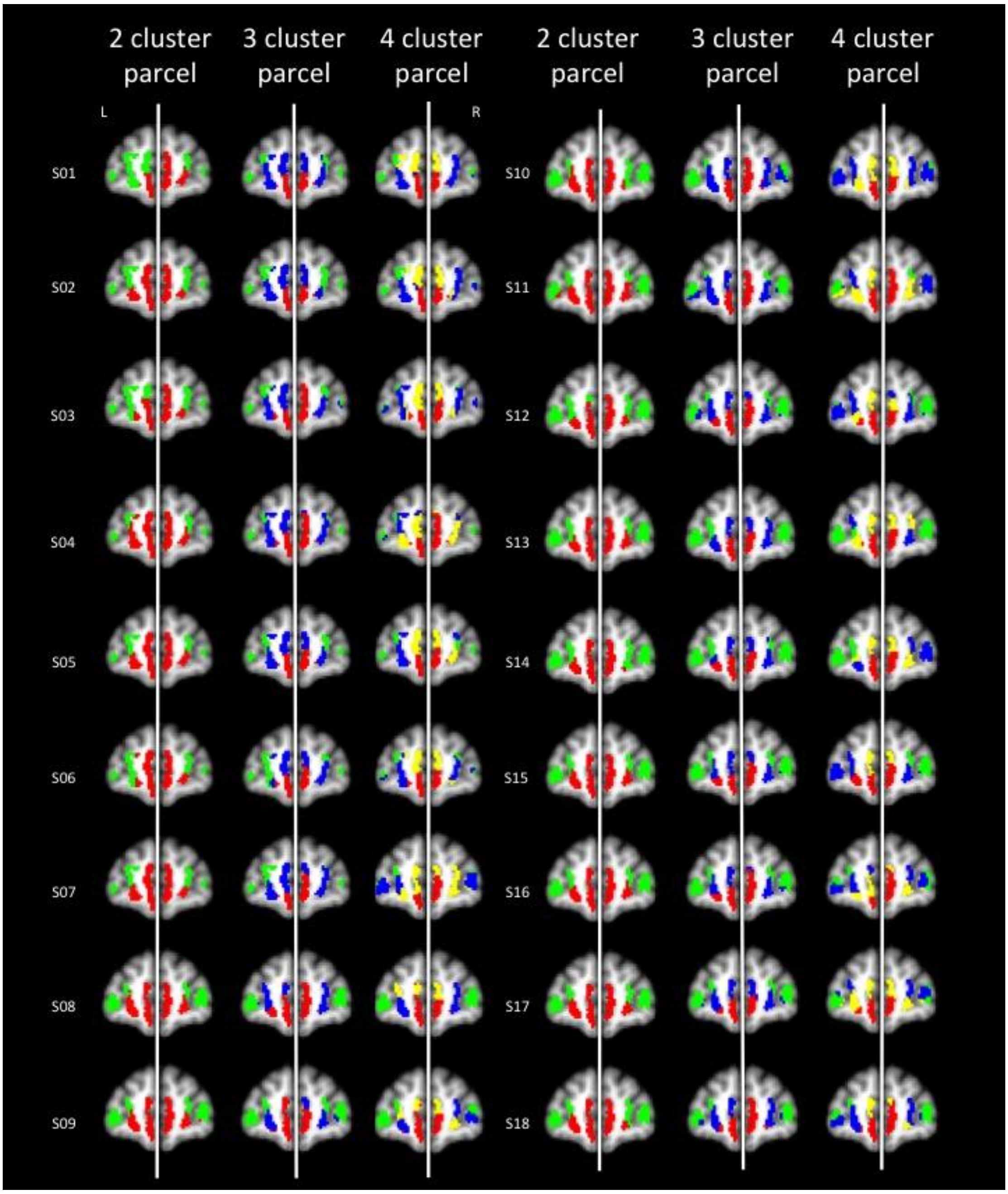
2 cluster parcel
3 cluster parcel
4 cluster parcel
2 cluster parcel
3 cluster parcel
4 cluster parcel
L
R
S01
S02
S03
S04
S05
S06
S07
S08
S09
S10
S11
S12
S13
S14
S15
S16
S17
S18

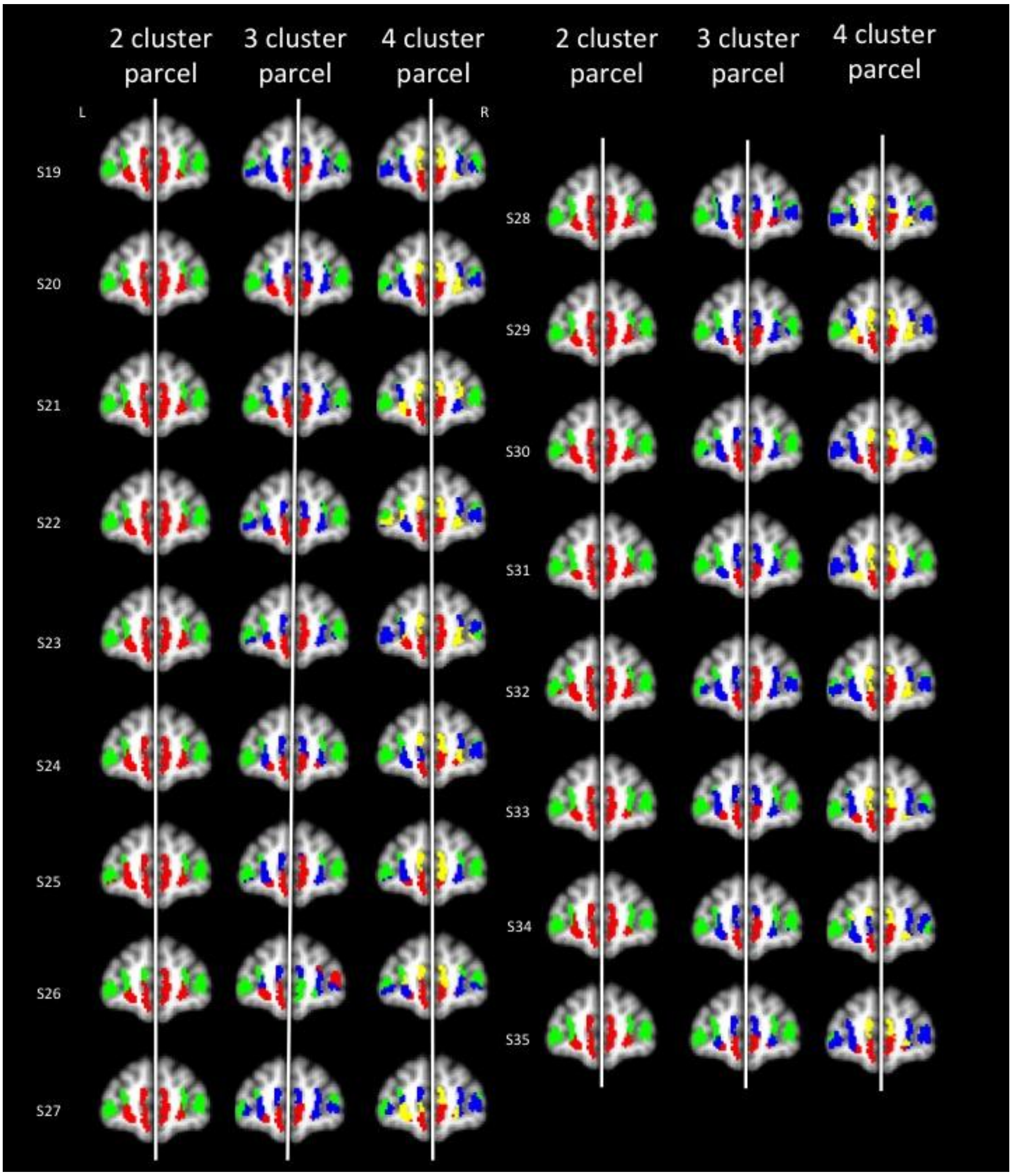

<u>Supplementary Figure 1:</u> Individual parcellation results for each subject, for each *K*-value. The lateral subregion is shown in green, the medial subregion in red. In the 3- and 4-subregion solutions, the rostral subregion is shown in blue. In the 4-subregion solution, the additional subregion is shown in yellow. Note the inconsistency of the yellow subregion across subjects. All parcellation results are shown on the MNI152 standard space brain at 1 mm resolution, at y = 56.

Supplementary Table 1: Peak MNI coordinates for regions exhibiting differential functional connectivity between subregions of the frontal polar cortex defined from the 2-subregion solution.

Seed Hemi	Contrast	Region	BA	Cluster Extent (mm^3)	Peak *T*-value	MNI (mm)		
						X	Y	Z
Right	Medial > Lateral	Medial Frontal pole	10	4394	15.76	8	60	-6
		Precuneus/Posterior cingulate cortex	7/23	1377	10.11	0	-58	26
		Middle temporal gyrus	21	249	7.36	-50	-12	-24
		Middle temporal gyrus	21	76	7.13	50	-4	-30
		Subgenual cingulate cortex	25	104	6.81	0	10	-18
		Parahippocampal gyrus/Amygdala	28	133	6.56	-26	-22	-22
		Middle temporal gyrus	21	19	6.37	66	-4	-18
		Parahippocampal gyrus	28	32	6.2	-20	-30	-12
		Posterior middle temporal gyrus	19	20	5.94	-46	-66	26
		Dorsolateral prefrontal cortex	8	15	5.83	-16	30	62
	Lateral > Medial	Lateral Frontal Pole	10	3263	19.76	36	50	14
		Lateral Frontal Pole	10	1576	10.58	-38	52	14
		Inferior Parietal lobule	40	1398	10.27	60	-36	48
		Dorsolateral prefrontal cortex	6	439	9.81	24	4	64
		Anterior Insula	13	422	9.26	-36	16	-4
		Ventrolateral Premotor cortex			7.5	-42	10	14
		Supplementary Motor cortex	6	577	9.12	6	18	48
		Anterior Insula	13	1122	8.26	34	18	-2
		Ventrolateral Premotor cortex	6		7.65	52	16	16
		Cerebellum (VI)		272	8.06	-28	-66	-28
		Inferior Parietal lobule	40	350	7.72	-58	-42	44
		Putamen		26	5.98	16	0	8
		Putamen			5.72	20	-10	14

Left	Medial > Lateral	Medial Frontal pole	10	2361	20.59	-8	58	-8
		Middle temporal gyrus	20	393	9.79	-56	-10	-24
		Dorsolateral prefrontal cortex	8	1242	8.41	-16	42	50
		Dorsomedial prefrontal cortex	8		8.03	10	50	44
		Precuneus/Posterior cingulate cortex	7/23	587	8.11	-2	-52	16
		Subgenual cingulate cortex	25	92	7.63	6	10	-18
		Parahippocampal gyrus	28	126	6.97	22	-16	-26
		Middle temporal gyrus	21	214	6.87	66	-4	-20
		Amygdala		180	6.77	-20	-6	-26
		Paracentral lobule	4	46	6.45	4	-32	72
	Lateral > Medial	Lateral Frontal Pole	10	3134	26.06	-36	50	12
		Inferior Parietal lobule	40	1144	11.16	56	-40	48
		Lateral Frontal Pole	10	2714	10.92	38	46	22
		Supplementary Motor cortex	6	736	10.39	0	22	48
		Anterior mid-cingulate cortex	32		6.24	10	24	32
		Anterior mid-cingulate cortex	24		5.75	10	26	20
		Anterior Insula	13	486	9.04	-34	16	-2
		Dorsolateral prefrontal cortex	6	251	8.32	-20	6	60
		Dorsolateral prefrontal cortex	6	354	7.96	32	2	60
		Ventrolateral Premotor cortex	6	510	7.51	48	10	12
		Anterior Insula	13		7.34	34	22	-2
		Inferior Parietal lobule	40	492	7.47	-46	-48	42
		Precuneus	19	59	7.09	12	-78	44
		Precuneus	19	49	6.48	-10	-68	52

Abbreviations: BA - Brodmann's Area; Hemi - Hemisphere.
